# Mouse Specific Cleavage-Resistant RAGE Splice Variant

**DOI:** 10.1371/journal.pone.0162120

**Published:** 2016-09-21

**Authors:** Tohru Fukai

**Affiliations:** Depts. of Medicine (Section of Cardiololgy) and Pharmacology, University of Illinois at Chicago, Chicago, Illinois, 60612, United States of America; Baker IDI Heart and Diabetes Institute, AUSTRALIA

Receptor for Advanced Glycation Endproducts (RAGE), a transmembrane, multi-ligand, pattern recognition receptor, has been implicated in a range of inflammatory disease including diabetes, cancers, and cardiovascular disease (CVD) [1]. In addition to the full length receptor, soluble form of RAGE (sRAGE) has been shown to prevent the development of numerous pathologic states, and therefore highlights RAGE as an attractive therapeutic target [2–7]. sRAGE lacks the trans-membrane domain and is secreted to the extracellular milieu [6]. RAGE and sRAGE share the entire extracellular portion encompassing the V, C1 and C2 immunoglobulin-like domains (Fig 1). This feature renders sRAGE to function as a decoy that binds ligands and reduces the inflammatory signaling capacity of its membrane-bound counterpart [5, 7]. The importance of sRAGE is underscored by clinical cohort studies showing that serum sRAGE levels are correlated with clinical disease states including CVD, diabetes, cancer and various inflammatory disease states [6, 8].

**Fig 1:**
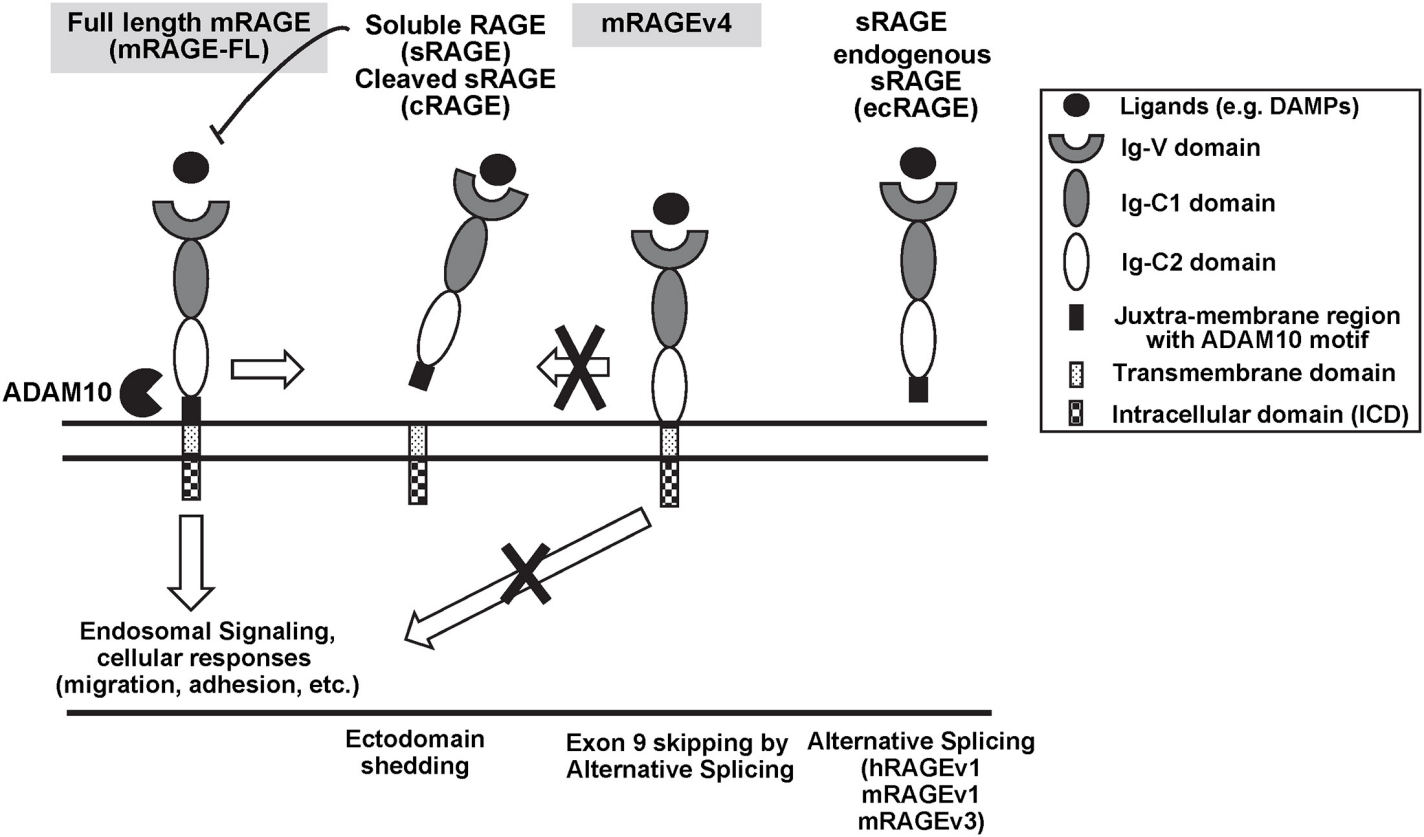
Proposed model of regulation and function of mouse specific cleavage-resistant RAGE Splice Variant mRAGEv4. RAGE is composed of the ectodomain (ECD)(three Ig domains: V, C1, and C2), juxtra-membrane region including ADAM motif, transmembrane domain, and intracellular domain (ICD). V and C1 domains carry a positive charge, in contrast to the C2 domain, which is negatively charged. Most ligands bind to the V domain of RAGE. RAGE can form soluble RAGE (sRAGE) by two possible mechanisms: the alternative splicing of the transmembrane domain (hRAGEv1, mRAGEv1, mRAGEv3) or the proteolytic cleavage from the cell surface (mRAGE-FL). Either in the baseline (constitutive response) or in the presence of stimuli, ADAM10 will be activated. This cleaves RAGE at the juxtamembrane region, releasing the RAGE ectodomain (ECD), which stimulates or inhibits endosomal signaling and various cellular responses (cell migration, adhesion, etc). The data about mouse-specific mRAGEv4 splice variant presented by Lin’s and Raucci’s group in this article suggests that the peptide motif recognized by ADAM10, which lacks in mRAGEv4, not only renders cleavage of mRAGE-FL by sheddases, but also directs the intracellular cellular trafficking of the full-length receptor.

Endogenous sRAGE isoforms have been identified in human and mouse serum which are generated through two distinct biological mechanisms (Fig 1). First, the alternative splicing of the transmembrane region of RAGE leading to a secreted isoform (endogenous sRAGE (esRAGE)) in both humans (variant 1 isoform) and mice (variant 1 and 3 isoforms). Second, the cleavage of extracellular domain (ectodomain or ECD) at the cell surface by ectodomain shedding. RAGE shedding is dependent on sheddases ADAM 10 and/or MMP-9 and can be induced with phorbol 12-myristate 13-acetate (PMA) and calcium ionophores, in a PKCα/β1 dependent manner [9–12]. Shedding-generated sRAGE (cleaved RAGE (cRAGE)) and esRAGE share the V/C1/C2 domains and presumably bind to the same ligands; the latter has a longer C-terminal sequence due to an alternative splicing-generated translational frame. It is unclear whether the two forms of sRAGE serve different functions in vivo. Ectodomain shedding of cell surface receptors can affect a number of processes such as the loss of cell-cell or cell-matrix interactions, the productions of a soluble ectodomain that acts as a agonist/antagonist for the cell surface protein and the release of the intercellular domain (ICD) of the receptor to induce cell signaling [13, 14].

Two articles in PLOS ONE by Lin’s and Raucci’s groups [15, 16] independently characterized the mouse-specific mRAGEv4 splice variant protein (mRAGE-v4), which is conserved in rodents but absent in primates and lacks 9 amino acids between the transmembrane and the Ig domains by alternative splicing of exon 9. **First**, RNA-Seq data confirm that mRAGEv4 is the most abundant RAGE mRNA isoform after full-length RAGE (mRAGE-FL) in mouse lung, while in heart all RAGE variants are almost undetectable. The proteins mRAGEv4 and mRAGE-FL are roughly equally abundant in mouse lung. This is consistent with the profiling of murine alternative splices performed by Hudson and colleagues [17]. **Second**, mRAGEv4 is resistant to constitutive shedding, in comparison to canonical full length mRAGE-FL. Lin’s group proved it by showing time-dependent constitutive shedding-generated sRAGE detected by immunoprecipitation and ELISA in the cell culture medium from A549 cells transfected with mRAGE-FL, but not with mRAGEv4 during 16 h cycloheximide chase. Raucci and colleagues showed that mRAGEv4 lacks the peptide motif recognized by both ADAM10 and MMP9, in particular, ADAM10. Of note, soluble RAGE isolated from murine lung ends at the C-terminus with Glu 328 [18], suggesting that in mice RAGE shedding predominantly via ADAM10, since the C-terminus produced by MMP9 proteolysis should end with Gly329. To support this, they found that the chemical ADAM10 inhibitor GI254023X or ADAM10 deficiency inhibits constitutive or stimulant (e.g. phorbol myristate acetate (PMA))-induced ectodomain shedding of mRAGE-FL, but not mRAGEv4 in rat alveolar type 1 like R3/1 cells and MEFs. **Third,** one of the striking features of mRAGEv4 is its exclusive localization to the plasma membrane. Consistent with previous reports [19–21], mRAGE-FL is not only localized at the plasma membrane, but also at the early and late endosome while mRAGEv4 is localized at the plasma membrane only. Furthermore, Lin and colleagues showed that after serum starvation, the majority of mRAGE-FL is localized at the lysosome, whereas mRAGEv4, in stark contrast, remains localized at the plasma membrane. This finding suggests that the 9-residue structural element which lacks in mRAGEv4 is not only required for cleavage of mRAGE-FL by sheddases, but also for the intracellular cellular trafficking of the full-length receptor. **Fourth**, there are splicing regulatory elements in the exon-intron structure of the mouse RAGE (Ager) gene, which are conserved in rat, but not in human and other primates. Thus, it may regulate the alternative splicing of the exon 9, resulting in mouse specific mRAGEv4.

The studies raised important questions about the regulation and functional role of mRAGEv4. **First**, impaired ectodomain shedding of mRAGEv4 may affect RAGE signaling, since the ectodomain cleavage of adhesion molecules has been shown to regulate not only their membrane expression but also cell migration, adhesion and activation [13, 14]. Indeed, Hudson and colleagues most recently showed that ectodomain shedding of RAGE is not only required for production of the soluble ECD to act as a decoy to inhibit RAGE signaling, but also required for gamma secretase-mediated release and subsequent processing of its intercellular domain (ICD), which contributes to RAGE-mediated cell migration and adhesion[22]. **Second**, although previous study was unable to detect sRAGE in murine serum using ELISA, Lin and colleagues showed that circulating murine sRAGE levels (~1000 pg/ml) appear to be higher than that of average normal human adults (range 500 to 900 pg/ml)[23]. Although the reason for this discrepancy is not clear, it may at least be due to differences in sRAGE measurement, mouse strain, gender, and age used in their studies. **Third**, impaired internalization of mRAGEv4 in the baseline and in response to stimulant may affect inflammatory signaling. Indeed, previous studies showed that the full-length RAGE is internalized to the endosome upon binding of pathogen DNA, whereby it dimerizes with Toll-like receptor 9 (TLR9) and mediates inflammatory signaling [20, 21] [24].

The data about mouse-specific mRAGEv4 splice variant presented by Lin’s and Raucci’s groups suggest that the peptide motif recognized by both ADAM10 and MMP9, which lacks in mRAGEv4, not only renders cleavage of mRAGEFL by sheddases, but also directs the intracellular cellular trafficking of the full-length receptor (Fig 1). There are many unanswered questions. What is the underlying molecular mechanism that regulates the differential cellular trafficking pattern of the two isoreceptors (i.e. mRAGE-FL and mRAGE-V4)? Does deletion of nine residues within the C2 ectodomain influence the overall folding of RAGE and hence affect its cellular behaviors? What is roles of mRAGE-FL and mRAGE-V4 in physiological and pathological status? What is the evolutionary and functional meaning of the variation in RAGE alternative splicing between rodents and humans? Addressing these questions will be essential to our understanding of the mechanism of inflammatory responses in which RAGE plays an essential role.

## References

[1] Kalea AZ, Schmidt AM, Hudson BI. Alternative splicing of RAGE: roles in biology and disease. Front Biosci (Landmark Ed). 2011;16:2756–70. Epub 2011/05/31. 21622207 10.2741/3884

[2] Bucciarelli LG, Wendt T, Qu W, Lu Y, Lalla E, Rong LL, et al RAGE blockade stabilizes established atherosclerosis in diabetic apolipoprotein E-null mice. Circulation. 2002;106(22):2827–35. 12451010 10.1161/01.cir.0000039325.03698.36

[3] Harja E, Bu DX, Hudson BI, Chang JS, Shen X, Hallam K, et al Vascular and inflammatory stresses mediate atherosclerosis via RAGE and its ligands in apoE-/- mice. J Clin Invest. 2008;118(1):183–94. 18079965 10.1172/JCI32703 PMC2129235

[4] Kalea AZ, See F, Harja E, Arriero M, Schmidt AM, Hudson BI. Alternatively spliced RAGEv1 inhibits tumorigenesis through suppression of JNK signaling. Cancer Res. 2010;70(13):5628–38. 10.1158/0008-5472.CAN-10-0595 20570900 PMC2919303

[5] Park L, Raman KG, Lee KJ, Lu Y, Ferran LJ Jr, Chow WS, et al Suppression of accelerated diabetic atherosclerosis by the soluble receptor for advanced glycation endproducts. Nat Med. 1998;4(9):1025–31. 9734395 10.1038/2012

[6] Schmidt AM. Soluble RAGEs—Prospects for treating & tracking metabolic and inflammatory disease. Vascul Pharmacol. 2015;72:1–8. 10.1016/j.vph.2015.06.011 26130225 PMC4547874

[7] Taguchi A, Blood DC, del Toro G, Canet A, Lee DC, Qu W, et al Blockade of RAGE-amphoterin signalling suppresses tumour growth and metastases. Nature. 2000;405(6784):354–60. 10830965 10.1038/35012626

[8] Ramasamy R, Yan SF, Schmidt AM. RAGE: therapeutic target and biomarker of the inflammatory response—the evidence mounts. J Leukoc Biol. 2009;86(3):505–12. 10.1189/jlb.0409230 19477910

[9] Galichet A, Weibel M, Heizmann CW. Calcium-regulated intramembrane proteolysis of the RAGE receptor. Biochem Biophys Res Commun. 2008;370(1):1–5. 10.1016/j.bbrc.2008.02.163 18355449

[10] Raucci A, Cugusi S, Antonelli A, Barabino SM, Monti L, Bierhaus A, et al A soluble form of the receptor for advanced glycation endproducts (RAGE) is produced by proteolytic cleavage of the membrane-bound form by the sheddase a disintegrin and metalloprotease 10 (ADAM10). FASEB J. 2008;22(10):3716–27. 10.1096/fj.08-109033 18603587

[11] Yamakawa N, Uchida T, Matthay MA, Makita K. Proteolytic release of the receptor for advanced glycation end products from in vitro and in situ alveolar epithelial cells. Am J Physiol Lung Cell Mol Physiol. 2011;300(4):L516–25. 10.1152/ajplung.00118.2010 21257730 PMC3075094

[12] Zhang L, Bukulin M, Kojro E, Roth A, Metz VV, Fahrenholz F, et al Receptor for advanced glycation end products is subjected to protein ectodomain shedding by metalloproteinases. J Biol Chem. 2008;283(51):35507–16. 10.1074/jbc.M806948200 18952609

[13] Garton KJ, Gough PJ, Raines EW. Emerging roles for ectodomain shedding in the regulation of inflammatory responses. J Leukoc Biol. 2006;79(6):1105–16. 16565325 10.1189/jlb.0106038

[14] Hayashida K, Bartlett AH, Chen Y, Park PW. Molecular and cellular mechanisms of ectodomain shedding. Anat Rec (Hoboken). 2010;293(6):925–37. 20503387 10.1002/ar.20757 PMC4621804

[15] Peng Y, Horwitz N, Lakatta EGa, Lin L. Mouse RAGE variant 4 is a dominant membrane receptor that does not shed to generate soluble RAGE. PLoS ONE. 2016:e153657. 10.1371/journal.pone.0153657 PMC5031407 27655067

[16] Di Maggio S, Gatti E, Liu J, Bertolotti M, Fritz G, Bianchi ME, et al The Mouse-specific Splice Variant mRAGE_v4 Encodes a Membrane-bound RAGE that is Resistant to Shedding and does not Contribute to the Production of Soluble RAGE. PLoS ONE. 2016:e153832. 10.1371/journal.pone.0153832 PMC5031469 27655137

[17] Hudson BI, Carter AM, Harja E, Kalea AZ, Arriero M, Yang H, et al Identification, classification, and expression of RAGE gene splice variants. FASEB J. 2008;22(5):1572–80. 18089847 10.1096/fj.07-9909com

[18] Hanford LE, Enghild JJ, Valnickova Z, Petersen SV, Schaefer LM, Schaefer TM, et al Purification and characterization of mouse soluble receptor for advanced glycation end products (sRAGE). J Biol Chem. 2004;279(48):50019–24. 15381690 10.1074/jbc.M409782200 PMC1868562

[19] Akirav EM, Preston-Hurlburt P, Garyu J, Henegariu O, Clynes R, Schmidt AM, et al RAGE expression in human T cells: a link between environmental factors and adaptive immune responses. PLoS One. 2012;7(4):e34698 10.1371/journal.pone.0034698 22509345 PMC3324532

[20] Tian J, Avalos AM, Mao SY, Chen B, Senthil K, Wu H, et al Toll-like receptor 9-dependent activation by DNA-containing immune complexes is mediated by HMGB1 and RAGE. Nat Immunol. 2007;8(5):487–96. 17417641 10.1038/ni1457

[21] Sirois CM, Jin T, Miller AL, Bertheloot D, Nakamura H, Horvath GL, et al RAGE is a nucleic acid receptor that promotes inflammatory responses to DNA. J Exp Med. 2013;210(11):2447–63. 10.1084/jem.20120201 24081950 PMC3804942

[22] Braley A, Kwak T, Jules J, Harja E, Landgraf R, Hudson BI. Regulation of RAGE ectodomain shedding and its role in cell function. J Biol Chem. 2016. 10.1074/jbc.M115.702399 PMC4933258 27022018

[23] Lazo M, Halushka MK, Shen L, Maruthur N, Rebholz CM, Rawlings AM, et al Soluble receptor for advanced glycation end products and the risk for incident heart failure: The Atherosclerosis Risk in Communities Study. Am Heart J. 2015;170(5):961–7. 10.1016/j.ahj.2015.08.008 26542505 PMC4638130

[24] Julian MW, Shao G, Bao S, Knoell DL, Papenfuss TL, VanGundy ZC, et al Mitochondrial transcription factor A serves as a danger signal by augmenting plasmacytoid dendritic cell responses to DNA. J Immunol. 2012;189(1):433–43. 10.4049/jimmunol.1101375 22675199 PMC3381894

